# Development of an Integrated Powered Hip and Microprocessor-Controlled Knee for a Hip–Knee–Ankle–Foot Prosthesis

**DOI:** 10.3390/bioengineering10050614

**Published:** 2023-05-19

**Authors:** Yousef Bader, David Langlois, Natalie Baddour, Edward D. Lemaire

**Affiliations:** 1 Department of Mechanical Engineering, University of Ottawa, 800 King Edward Ave., Ottawa, ON K1N 6N5, Canada; nbaddour@uottawa.ca (N.B.); elemaire@ohri.ca (E.D.L.); 2 Össur, Grjothals 1–5, 110 Reykjavik, Iceland; dlanglois@ossur.com; 3 Faculty of Medicine, University of Ottawa, 451 Smyth Road, Ottawa, ON K1H 8M5, Canada

**Keywords:** hip–knee–ankle–foot prosthesis, hip amputation, microprocessor-controlled prosthesis, powered hip, integrated hip knee

## Abstract

Hip–knee–ankle–foot prostheses (HKAF) are full lower-limb devices for people with hip amputations that enable individuals to regain their mobility and move freely within their chosen environment. HKAFs typically have high rejection rates among users, as well as gait asymmetry, increased trunk anterior–posterior lean, and increased pelvic tilt. A novel integrated hip–knee (IHK) unit was designed and evaluated to address the limitations of existing solutions. This IHK combines powered hip and microprocessor-controlled knee joints into one structure, with shared electronics, sensors, and batteries. The unit is also adjustable to user leg length and alignment. ISO-10328:2016 standard mechanical proof load testing demonstrated acceptable structural safety and rigidity. Successful functional testing involved three able-bodied participants walking with the IHK in a hip prosthesis simulator. Hip, knee, and pelvic tilt angles were recorded and stride parameters were analyzed from video recordings. Participants were able to walk independently using the IHK and data showed that participants used different walking strategies. Future development of the thigh unit should include completion of a synergistic gait control system, improved battery-holding mechanism, and amputee user testing.

## 1. Introduction

Hip–knee–ankle–foot prostheses (HKAF) help people with hip disarticulation (HD: full amputation of the lower limb) and hemipelvectomy (HP: full amputation of the lower limb and partial amputation of the pelvis) move within their chosen environment. HKAF are the most complex prostheses because they must include hip, knee, and ankle joints that function together to enable the user to move with little restriction. Prostheses enable amputees to regain their mobility; however, one in three HKAF prostheses are rejected by their user [1,2]. Reasons for rejection include slow walking speed, need for secondary walking aids [1], prosthesis weight, difficulty of use [3], and increased energy expenditure compared to the use of a wheelchair [4].

Recent prosthesis development has focused on integrating microprocessor-controlled (MPC) joints and control systems to offer real-time gait adjustments to achieve optimal walking [5,6,7]. Commercially available MPC prostheses utilize passive joints with variable damping to control joint acceleration [8] or active joints with electric motors to provide torque for tasks such as stair climbing and standing from a sitting position [9]. MPC prostheses can provide advanced control over the joint by collecting sensor data to determine user intention and adapt to the user’s environment. Additionally, MPC devices can improve user safety with features such as stumble recovery [9].

Commercial passive and active MPC knee [9] and ankle [10] prostheses are available for transfemoral and transtibial amputees. Transfemoral amputees transitioning from mechanical to MPC knee joints showed improved balance and stability [11] and increased daily activity and satisfaction [12], while transtibial amputees transitioning to MPC ankle joints showed an improved ability to adapt to different walking environments such as inclines [13,14]. Non-MPC hip prostheses are commercially available; however, there are currently no commercially available MPC hip prostheses and only one research MPC hip device [15].

Several studies have reported asymmetrical gait when walking with a mechanical hip prosthesis on flat level ground. Karimi et al. [16] showed large differences in range of motion (ROM) between the intact and prosthetic limbs of amputees using mechanical hip and knee joints. Gait asymmetry was still observed when an MPC knee joint was used with a mechanical hip joint; for example, intact leg stance phase of 72% of the stride and prosthetic stance of 57% [17]. When compared to able-bodied gait, trunk anterior–posterior lean was greater than normal, trunk lateral flexion shifted towards the intact limb, and pelvic anterior–posterior motion was greater during early swing [17,18]. Amputee pelvic tilt ROM was 21° higher than able-bodied controls when using a 7E7 mechanical hip joint, and 16° higher with an Ottobock Helix3D mechanical hip joint. Increased pelvic ROM was attributed to the excessive hip swing to increase hip flexion during gait [18].

Ideally, an MPC hip joint would also use an MPC knee joint; therefore, the opportunity to combine both joints into a single prosthetic thigh unit arises. Currently, few prostheses combine more than one joint together to form an all-in-one device. Commercially, the only multi-joint prosthesis available to amputees is the Blatchford Linx, a MPC knee–ankle–foot prosthesis that provides transfemoral amputees with hydraulically controlled knee and ankle joints that act synergistically to enable walking, standing, slope and stair descent, stand-to-sit, and stumble recovery [19]. The only MPC HKAF device was built by Ueyama et al. [15], using DC motors positioned within the thigh to power the hip and knee joints. The hip and knee joint shared microcontroller boards, a battery, and sensor data, reducing the required hardware. Walking tests showed that it is possible to closely mimic able-bodied hip and knee gait patterns when using DC motors to actuate the hip and knee joints; however, the device was not self-contained and required users to wear a belt that contained the battery. The prosthesis was also too large to fit under clothing.

An active MPC hip prostheses should generate hip torque to assist with swing initiation, provide increased hip flexion while walking on slopes, and improve extension/flexion timing. Integrating the hip and knee joints into a self-contained, all-in-one system allows the two joints to share resources such as batteries, electronics, and sensors, which is more efficient than having two independent joints for the hip and knee. Sharing sensor data and control electronics allows for future development of advanced control strategies that can enable joint co-ordination during gait and enhanced sit-to-stand motion.

The goal of the research completed in this paper was to develop a prototype MPC thigh component for a powered HKAF prosthesis and evaluate the prototype’s mechanical performance for level-ground walking. The integrated hip–knee (IHK) unit must include a powered hip joint, MPC knee, and all required electronics and sensors to operate the device. Ultimately, a MPC HKAF prosthesis for hip amputees should enable users to safely move within their day-to-day lives with little restriction.

## 2. Materials and Methods

### 2.1. Design Requirements

The following design requirements influenced IHK shape, size, and testing protocols:

- The thigh unit must accommodate people with different thigh lengths. Based on US anthropometric data, the device must accommodate a minimum thigh length of 31.9 cm [20]. Discussions with a prosthetist determined that 10 cm can be added to the minimum thigh length as a result of the hip joint mounting to the front of the socket;
- The device must integrate a motor-powered hip joint and a variable-resistance passive MPC knee joint;
- The device must be modular to allow for modifications to accommodate different hip joints;
- The prosthesis weight must be minimized to reduce likelihood of rejection [3]. The IHK mass should be similar to the combined weight of the Össur Power Knee (3.2 kg) [21] and the Össur Rheo Knee 3 (1.6 kg) [22] (i.e., 4.8 kg);
- The hip and knee joints must be electronically integrated to share sensor data, battery power, and processing power;
- Device should fit under pants and other clothing;
- The device structure must withstand typical walking loads without failing;
- The mechanical structure must protect the electronics from typical loads experienced during walking.

### 2.2. System Design

**Figure 1:**
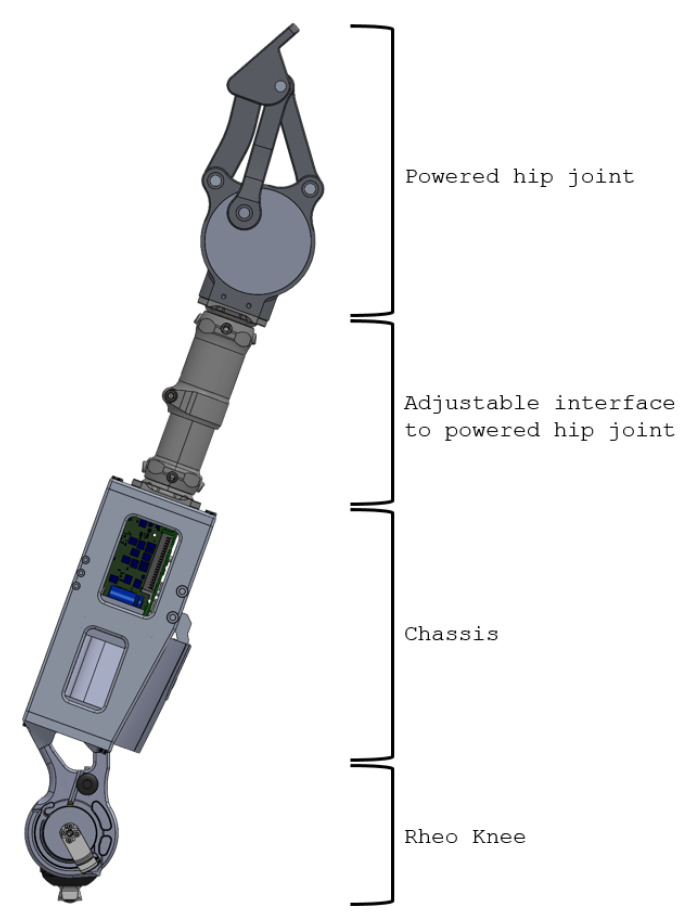
IHK unit consisting of a powered hip, chassis, interface to connect the powered hip to the chassis, and a Rheo Knee 3 actuator. The interface shown includes two male pyramid adapters and an Össur height-adjustable pylon.

The complete IHK unit (Figure 1) consists of four components:

- Powered hip joint: The most proximal part of the thigh unit is the powered hip joint that is positioned anteriorly to the pelvis and is actuated by a DC motor. The thigh unit is designed to accept different powered hip joint devices that attach to the remaining HKAF components through a pyramid adapter or pylon. The hip joint used in this research has a male pyramid adapter on its distal end, which is used to connect to the adjustable interface;
- Adjustable interface: The adjustable interface is constructed of industry standard prosthetic-interfacing components such as male and female pyramid adapters and pylons to allow length and angular adjustment between the chassis and the powered hip joint. The adjustable interface in this example connects to the powered hip joint’s male pyramid adapter using a female pyramid adapter. The distal end of the adjustable interface can use male or female pyramid adapters or a pylon clamp, depending on the person’s needs and decisions by the prosthetist;
- Chassis: The main structural component of the design that contains and protects electronics, sensors, and battery used to power and control the Rheo Knee 3 actuator and the powered hip joint. A standard four-hole pyramid adapter can be attached on the proximal end of the chassis using four bolts to connect to the adjustable interface, and it has the required mounting points to align and attach to the Rheo Knee 3 actuator on the distal end of the chassis;
- Rheo Knee: The distal part of the IHK unit is the Rheo Knee 3 actuator oriented upside down so that the proximal surface can screw into the designed aluminum chassis’ mounting points and the male pyramid adapter can interface with the shank components.

The scope of this research covers structural design and testing of the IHK unit chassis and integration of a knee actuator. The powered hip joint design, electronics, and software were developed and unit-tested in parallel research and will not be discussed in detail.

### 2.3. Chassis Design

**Figure 2:**
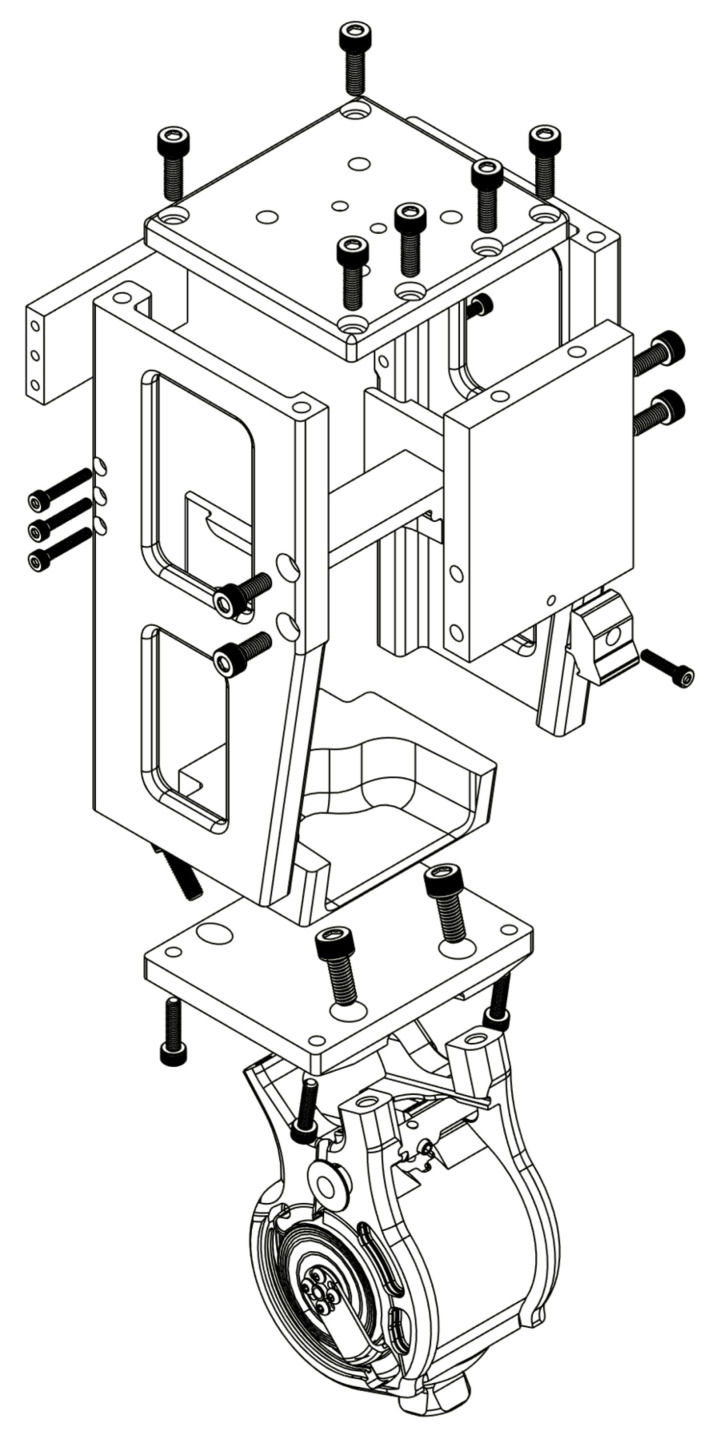
IHK chassis design.

Six aluminum parts were assembled using steel bolts to create the chassis structure (Figure 2).

Aluminum 2024 was used to balance chassis mass, strength, and manufacturability. The left and right plates are the primary load-bearing components that connect all other chassis components together, and protect the electronics. Two cut-outs were made in each side plate to reduce device weight and provide access to the electronics compartment. The front and back supports connect the two side plates and provide torsion resistance to stop the chassis from excessively twisting when subjected to torsion loads. The front and rear supports also reinforce the two side plates from bowing when under high compressive load. The top plate was designed to accept industry standard male and female four-hole pyramid adapters.

Four protrusions on the bottom plate align with the Rheo Knee 3 mounting points, securing the joint to the chassis. After consultations with a prosthetist, the actuator mounting points were designed to mount the knee at 20° relative to the chassis vertical axis. Since the hip joint mounts to the front of the pelvis, the IHK chassis would be rotated 20 degrees anterior to the pelvis; therefore, the actuator mounting points angle the actuator to allow it to operate in an upright/vertical orientation underneath the pelvis, similar to its orientation in the Rheo Knee 3.

Three 3D-printed plastic components were designed to hold the battery in the lower half of the chassis, leaving the top half of the chassis free for the electronic components. The top and bottom battery support components tightly position the battery within the chassis to align battery connection pins to the electronics, but are not load-bearing. The battery lock engages with a tab in the battery to keep the battery in the device. The chassis is designed such that loads applied to the aluminum chassis components are not transmitted to the plastic components; therefore, PLA plastic was chosen for these components due to its lower toxicity compared to other plastic filaments and its higher stiffness to keep the battery correctly in place. 

### 2.4. IHK User Adjustability

The chassis must accommodate a wide range of adjustments to allow compatibility with as many users as possible. Two levels of adjustability were developed for the chassis: the adjustable interface and customized chassis modifications.

#### 2.4.1. Adjustable Interface

The primary purpose of the adjustable interface is to connect the chassis top plate to the powered hip joint using industry standard prosthetic components, such as male and female pyramid adapters and pylons. Pyramid adapters are industry standard connectors used to connect prosthetic components together and they provide ±10° of angle adjustability between the components they connect. Pylons are hollow metal tubes that add length between prosthetic components. Pylons are normally cut to length by a prosthetist or technician to the required size for the patient. Pylons connect to other prosthetic components using a pylon clamp, which attach pyramid adapters to the ends of the pylon.

Different adapter combinations can be used by a prosthetist to adjust and align the IHK to the user. The prototype powered hip joint used for this research was 13.9 cm from hip center of rotation to the most distal point of the joint. The prototype chassis was 25.1 cm from knee center of rotation to the top plate surface. To meet the length design requirements, the minimum configurable length of the adjustable interface must be 3 cm or less. Since commercial implementations of this hardware would be smaller, more adjustability for shorter thigh dimensions is expected following the initial prototype phase. Different potential configurations of the adjustable interface are shown in Table 1.

#### 2.4.2. Customized Chassis Modifications

**Figure 3:**
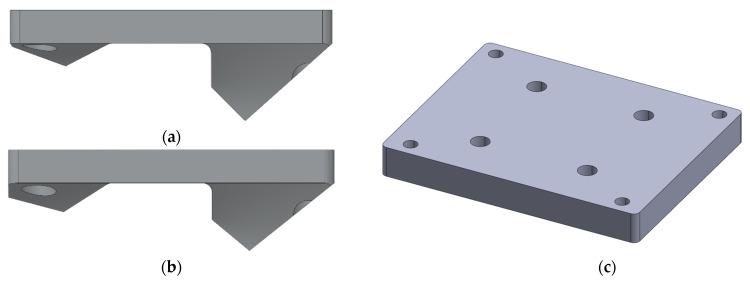
Example of different chassis bottom plate designs: (**a**) standard 20° Rheo Knee actuator bottom plate; (**b**) modified 15° Rheo Knee actuator bottom plate; and (**c**) bottom plate designed to accept a four-hole pyramid adapter.

Prosthetists can choose customized top and bottom plates to accommodate non-standard connections with other prosthetic components (Figure 3). Customization is enabled by the modular chassis design where individual parts can be removed without fully disassembling the chassis. For example, multiple versions of the chassis bottom plate were designed and manufactured to test different knee actuator configurations during user testing. These bottom plates include the standard 20° angle, a 15° angle plate, and a bottom plate that accepts a four-hole pyramid adapter instead of direct joint mounting.

**Figure 4:**
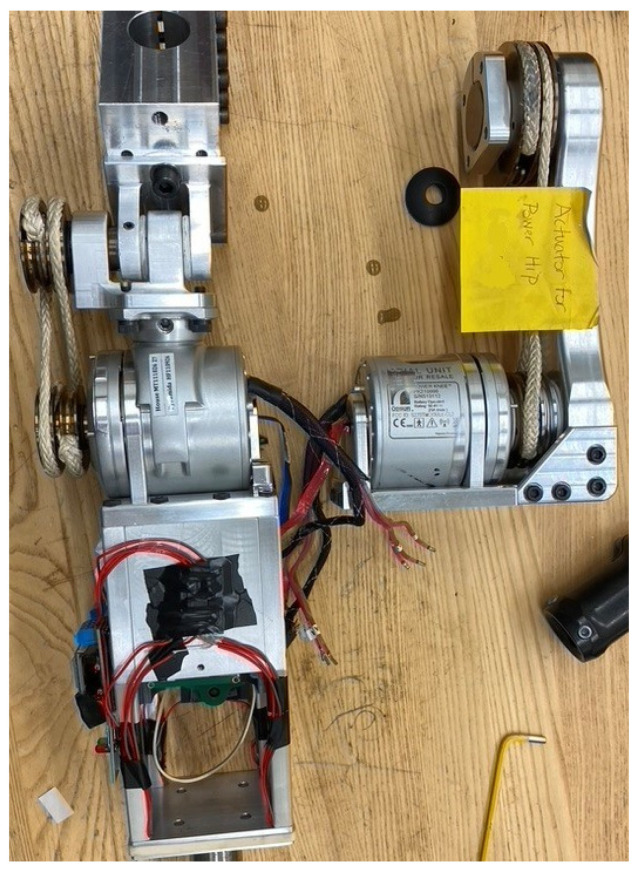
Example of two custom plates designed to directly connect powered hip joints to the top of the chassis, removing the adjustable interface.

Similarly, chassis top plates were designed to interface directly with the motor of some hip joint designs instead of using the original adjustable interface (Figure 4). These top-plate modifications allowed further reduction of the IHK length; however, this results in the loss of adjustability between the chassis and the powered hip joint.

### 2.5. Bench-Testing Preparation

Based on ISO-10328:2016 [24], the IHK chassis was classified as “Complete structure of transfemoral/knee-disarticulation prosthesis or distal part of hip-disarticulation prosthesis without foot unit”. The required tests for this category include principal structural tests, tests in torsion, test in maximum knee flexion, and tests on knee locks. Tests on maximum knee flexion and knee locks are knee actuator tests rather than tests on the IHK chassis. Since the IHK used the tested Össur Rheo Knee, only the principal structural tests and tests in torsion were completed.

**Figure 5:**
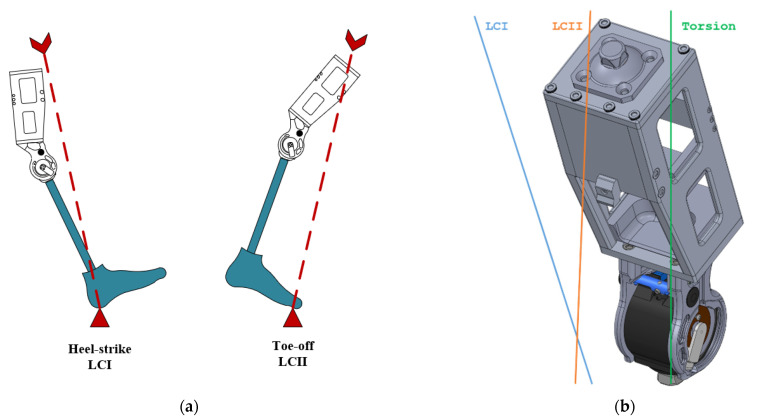
Loading conditions as defined by ISO-10328:2016 [24]: (**a**) loading condition load lines represented as stages of the gait cycle; and (**b**) loading condition load lines as applied to the chassis.

The principal structural tests included proof load, ultimate static load, and cyclic fatigue tests. Principal structural tests were conducted under two different loading conditions that simulated the most extreme loading conditions during gait: loading condition I (LCI) at heel strike and loading condition II (LCII) at toe-off (Figure 5). Loading levels are defined by the testing specifications.

The prosthesis classification, test terminology, test loads, and test procedure were copied by Yousef Bader with the permission of the Standards Council of Canada (SCC) on behalf of ISO. The standard can be purchased from the national ISO member in your country or the ISO Store. Copyright remains with ISO.

Based on preliminary simulation results, only P5 load-level testing conducted in LCI loading condition was considered for bench testing (i.e., highest load level for heavier people). Since the prototype chassis was needed for further IHK testing, destructive testing was not performed on the chassis. Based on this decision, only LCI P5 proof-loading tests, as defined by ISO-10328:2016 (Table 2), was completed.

**Figure 6:**
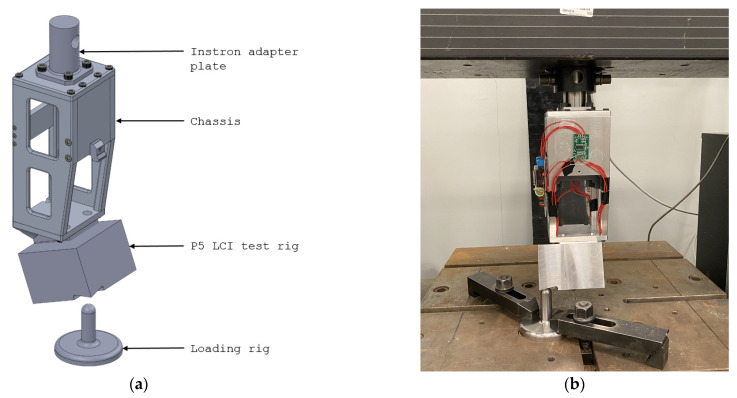
Bench testing setup of the chassis using an Instron 4482 test machine: (**a**) test components for bench testing; and (**b**) chassis mounted to the test hardware.

Bench testing was conducted using an Instron 4482 machine to apply loads onto the chassis. An adapter plate was designed and attached to the top moving arm using the pyramid adapter mounting holes on the top plate. The P5 LCI test rig component was designed to replace the Rheo Knee in bench testing. The component had the same mounting holes as the Rheo Knee, and had a machined loading surface that allowed loads to be applied along the LCI loading line. A loading rig was manufactured with a long cylindrical structure with a semi-spherical surface at the tip. The loading rig was placed beneath the load application surface of the P5 LCI test rig and secured using T-clamps (Figure 6). The procedure from ISO-10328:2016 proof load tests was used to test the chassis:

1. Lower the chassis slowly until a 1024 N settling load is applied. The load is held for more than 10 s and less than 30 s;
2. Raise the Instron crosshead until no force is applied to the chassis. Let the chassis rest for a period between 10–20 min before continuing;
3. Move the chassis downwards, loading 100–250 N/s until the applied load reaches the required proof load value. Maintain the applied load for 30 (±3) s;
4. Raise the chassis until it is no longer in contact with the loading rig.

### 2.6. User-Testing Preparation

Device evaluation was completed using a hip prosthesis simulator that allowed able-bodied people to walk on an HKAF prosthesis [25]. The test protocol was approved by the University of Ottawa Research Ethics Board, ethics file number H-08-21-7062.

#### 2.6.1. Test Prosthesis

**Figure 7:**
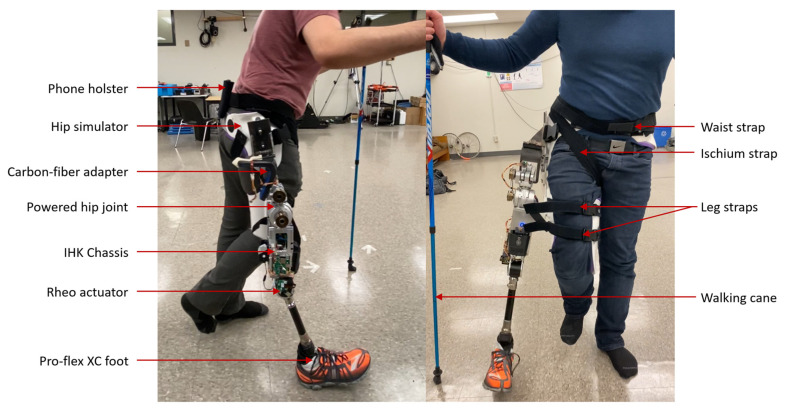
Test prosthesis setup attached to hip simulator.

The test prosthesis consisted of a prototype IHK with a front-mounted powered hip joint attached directly to the top of the chassis using a custom top-plate mount. A pylon connected an Össur Pro-Flex XC foot [26] to the IHK to complete the HKAF prosthesis. The simulator was secured to the participant using two straps around the leg, one strap around the waist to tighten the pelvic basket, and one strap underneath the right ischium. A phone holster was secured above the simulator to position a smartphone on the pelvis for walking data recording (Figure 7).

The hip–knee electronics were still in development at the time of testing; therefore, separate microprocessors and batteries were included for each joint. As a result, some of the electronics did not fit in the dedicated electronics chamber within the chassis and had to be secured to the exterior of the chassis. Test prosthesis mass is shown in Table 3.

#### 2.6.2. Participants

For safety, only able-bodied individuals participated in preliminary test trials. Three male participants volunteered to test IHK functionality (Table 4). Each participant had previous walking experience on the hip simulator with the powered hip joint assembled with an Össur Rheo Knee 3 and an Össur Pro-Flex XC foot, as well as an Ottobock Helix3D hip joint assembled with an Össur Rheo Knee 3 and an Össur Pro-Flex XC foot. Participants had the option of using one or two walking canes. Participant A had the most experience walking on the hip simulator.

#### 2.6.3. Control System

**Figure 8:**
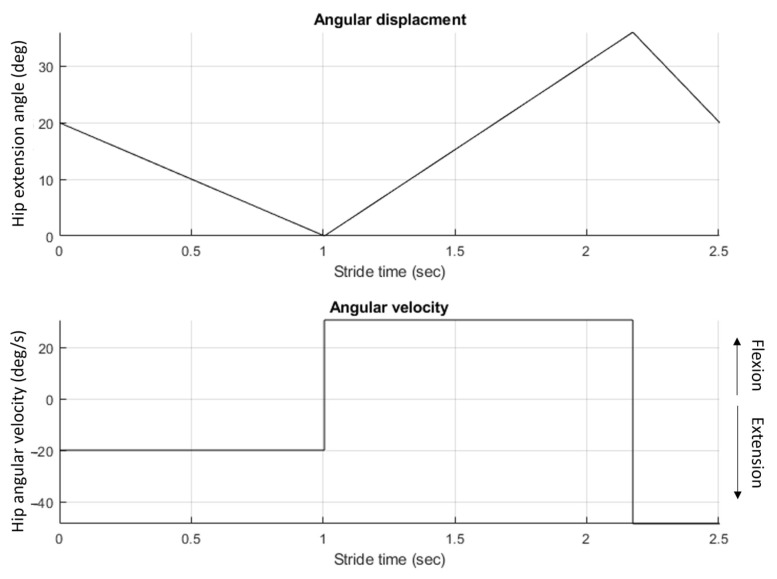
Hip angle and angular velocity profiles used for the IHK testing.

At the time of testing, the gait intent recognition algorithm was still in development; therefore, the hip joint was programmed to follow a pre-defined joint angular velocity profile that mimicked level walking (Figure 8). After undergoing tuning and training sessions with the IHK, the hip angle trajectory was programmed to have a 0° maximum extension and a 36° maximum flexion for a total ROM of 36° over 2.5 s, resulting in a cadence of 24 steps/min.

The gait profile was uploaded into the hip joint control software, which controlled the hip joint to follow the defined gait profile. A laptop connected to the IHK through Wi-Fi and Bluetooth initiated and stopped data collection and powered hip motion. The knee used a Rheo Knee 3 control system with minor modifications since load inputs were from strain gauges applied to the chassis instead of typical shank forces. Initial feedback from the participants showed that the knee was not entering swing phase mode and remained locked in extension throughout swing phase; therefore, further changes were made to the control system to make stance detection less sensitive and allow the knee to enter swing phase and flex properly.

#### 2.6.4. Data Collection

Using the TOHRC data-logger application [27], a smartphone (Google Pixel 4a (2020)) was secured to the participant’s lower back using a strap and holster to collect pelvic tilt and pelvic obliquity data at a 60 Hz. Videos of the test sessions were recorded at 60 frames per second using an iPhone 11 Pro (2019) while keeping the phone parallel to the prosthesis and having both the HKAF and the contralateral limb visible throughout the tests. Videos were analyzed using Kinovea analysis software (version 0.9.5) [28] to determine timestamps for foot-strikes and foot-offs for the prosthetic and contralateral limbs. Hip and knee angles were collected at 50 Hz from joint Hall-effect sensors built into the joint actuators.

Video recordings and hip angle and knee angle data were synchronized during terminal swing at the first stride of each test. The data point and video frame when the hip stopped flexing were used for hip synchronization. Knee data were aligned with video at the frame when the knee stopped extending. Eight consecutive strides were isolated and evaluated for each participant.

## 3. Results

### 3.1. Bench-Testing Results

No permanent deformation or bending was observed on any of the chassis components, and none of the threaded holes appeared to be damaged after testing. By using the Instron displacement measurement readout, it was possible to determine if the chassis had experienced permanent deflection by measuring the displacement at which the chassis came in contact with the loading rig before and after proof load. The chassis did not experience noticeable permanent deflection after proof loading.

### 3.2. User-Testing Results

No mechanical issues occurred over the three separate tuning, practice, and data recording sessions with the HKAF prosthesis simulator. The electronics and control system used in this evaluation were sufficient to demonstrate the appropriate mechanical system function; however, a pre-commercial version will require optimized electronics and the implementation of an adaptive control system.

Table 5 shows stride parameter results from eight consecutive strides for each participant. Average stride time for all participants was 2.47 ± 0.14 s and cadence was 24.35 ± 1.41 steps per minute. These values are within the range of programmed values that were loaded into the gait profile, which were 2.5 s stride time and 24-steps-per-minute cadence.

Table 6 shows hip and knee angle ROM. The IHK hip average ROM was 36.57 ± 3.23° with an average hip extension of −5.16 ± 4.98° and an average hip flexion of 31.41 ± 2.44°. The ROM met the programmed gait profile value of 36°, while hip extension (programmed at 0°) and flexion (programmed at 36°) values did not.

**Figure 9:**
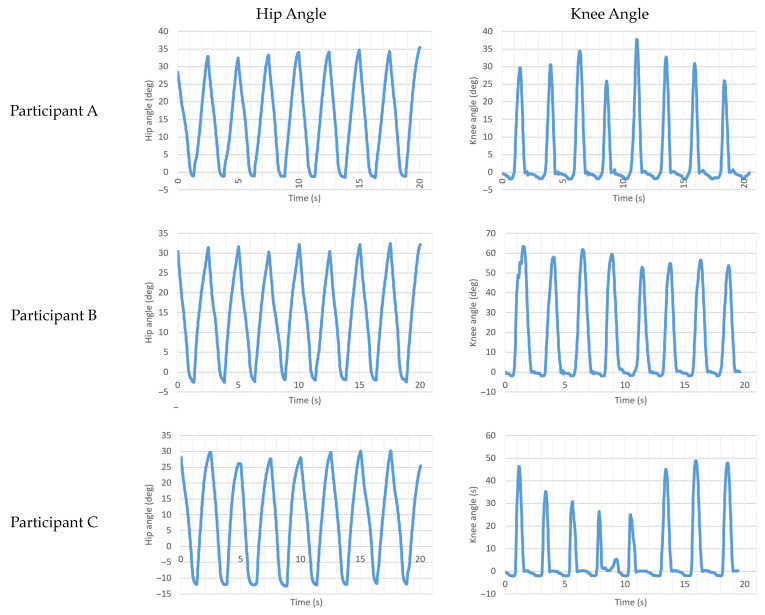
Hip angle versus time and knee angle versus time for eight consecutive steps for Participants A, B, and C.

Hip angle versus time and knee angle versus time for all three participants are shown in Figure 9. Note that Participant C had a slight stumble during his fourth step, which is visible in the knee angle versus time graph.

Pelvic tilt and pelvic obliquity data were collected using a smartphone placed on the participant’s rear pelvic region. An average pelvic tilt range of 16.02 ± 3.40° was observed across participants, with Participant A having the smallest range of 14.26 ± 2.07°.

## 4. Discussion

The mechanical and walking tests demonstrated that the IHK unit successfully provides an integrated-powered-hip-and-microprocessor-controlled-knee approach for hip-level amputees. The integrated thigh unit was evaluated to study the mechanical aspect of this device within a HKAF prosthesis. The chassis design was guided by a set of criteria based on anthropometric data, prosthetist guidance, and safety standards for lower limb prostheses. The defined safety and structural stability criteria were successfully met, but further electronics and control system development is required before moving to pre-commercial device evaluation.

The IHK successfully met the minimum configurable length requirement, integrated a motor-powered hip joint and microprocessor-controlled knee joint, allowed prosthetist configuration and customization, and passed ISO-10328:2016 proof load testing requirements. The total IHK weight was 5 kg, which was 200 g greater than the design criteria. As an initial prototype, the device weight should decrease as the design is optimized. Examples of optimization include using a single shared battery (two batteries were used in this prototype) and a single shared microprocessor for all IHK functionality (three microprocessors were used during testing, one for each joint and one for load sensing).

The chassis was disassembled and analyzed post-bench testing to determine the state of the components. An analysis of the chassis components revealed no visible signs of mechanical yield, and showed that the chassis could sustain expected loads during walking without mechanical failure.

An analysis of stride and swing time showed that participants employed different walking strategies. Participant A had a longer prosthetic stance time (1.53 ± 0.11 s, 64% of stride) compared to Participants B (1.22 ± 0.11 s, 49%) and C (1.26 ± 0.04 s, 51%) (i.e., Participant A spent more time weight bearing on the prosthesis). This was also reflected by Participant A having 1.20 ± 0.12 s double support time compared to Participants B (0.94 ± 0.07 s) and C (0.78 ± 0.07 s). Comparing the prosthetic and intact limbs, gait was asymmetrical for all participants with an average HKAF step time of 1.34 ± 0.16 s compared to 2.12 ± 0.15 s for the intact limb. Swing phase analysis showed gait asymmetry with the HKAF having an average swing time of 1.15 ± 0.22 s and the intact limb average swing time of 0.35 ± 0.11 s. Gait asymmetry was attributed to the use of a predefined gait profile for all three participants instead of an adaptive gait control system. The results showed that the participants had some influence on step and swing speeds even with a static gait profile. During stance, Participants B and C chose to use their body weight to force the hip to move faster and end the stance phase earlier, while Participant A chose to initiate heel contact earlier than the gait profile was programmed, to have a small period to prepare for the next stride before the gait profile entered the stance phase.

An analysis of the IHK hip and knee ROM showed that the programmed hip range of motion was met but programmed hip extension and flexion values were not. This discrepancy may be caused by the control system undershooting the swing phase motion or participants forcing the hip to move in a motion they found more comfortable, which was enabled by the backdriveable motor. Another potential cause for the flexion deficiency is that toe drag was observed during swing, which would have produced resistance to flexion during swing. Video analysis showed that Participants B and C had difficulty achieving toe clearance during swing, which explains their results not matching the programmed hip ROM value. These results also showed that Participant C had a different walking strategy than the other participants, since he chose to force the hip up to −12.49° of extension compared to −1.58° for Participant A and −2.55° for Participant B. This is believed to be caused by Participant C choosing a gait strategy that he found more comfortable at the time of testing. It is believed that Participant A experienced little toe drag due to his experience walking on the hip simulator, allowing him to correctly make the gait adjustments to allow the foot to clear the ground.

A potential cause of toe drag is that the knee control system is providing too much resistance or maintaining resistance for too long into knee flexion during early swing. This would cause insufficient knee flexion for toe clearance during swing, resulting in the observed toe drag. This can be adjusted and tuned in future revisions of the IHK control system. Another potential cause of the toe drag is by the Rheo Knee actuator extension spring, which is a torsion spring built into the Rheo Knee that is designed to extend the knee under no-load conditions. The Rheo Knee extension spring was not modified, and was designed with the assumption that the Rheo Knee electronics and battery would be located in the shank, making the shank heavier. Since the IHK shifts the knee electronics and battery weight to the thigh, the shank is lighter and the torsion spring may provide too much extension torque for the dampened knee movement, causing the toe to drag on the floor.

IHK synergistic gait control could potentially result in better movement across a variety of mobility scenarios. The chassis design facilitates hip and knee units working together by providing the infrastructure for shared sensor input, electronics, and battery power. In the current prototype, only chassis load data were shared between the knee and hip control systems. Future IHK development should focus on hip and knee joint control integration to allow for unrestricted sensor-data sharing and synergistic control.

The mechanical tests and functionality tests with able-bodied participants demonstrated that the powered HKAF prosthesis is safe for next-phase evaluation with HD or HP amputees. The goal of this research was to test the IHK mechanical aspects when used for level walking; however, data from able-bodied participants wearing a hip simulator that positions the HKAF prosthesis laterally to the pelvis instead of directly in front of the pelvis may differ from people with hip-level amputations and a good fitting prosthesis. Mounting the IHK to the hip simulator is not as rigid as direct mounting to a socket; therefore, some movement of the prosthesis relative to the pelvis may occur as the hip simulator stretches and flexes while under load. Additionally, the lateral mounting point results in the prosthesis not being directly below the body center of mass, creating a cantilever effect on the prosthesis. As a result, the loads applied to the IHK could differ from loads expected by amputee users. The laterally mounted HKAF may also result in able-bodied users developing different gait strategies than amputee users, to compensate for differences between the simulator and socket systems. 

Table 7 provides a brief comparison between the IHK and alternative HKAFs for hip amputees. 

Patient-reported outcome measures (PROM) are commonly used to measure the performance of rehabilitation procedures. Since this study had able-bodied participants using a hip simulator, PROMs were not used since they would not be comparable to other studies that included amputee participants. Future work will involve testing with amputee participants, after the adaptive gait control system has been completed. Future work involving amputee participants should also include PROMs to allow comparisons between the IHK and other HKAF prostheses.

## 5. Conclusions

An integrated thigh unit was developed that combined a powered hip and microprocessor-controlled knee. Device structural safety was tested through bench testing on an Instron test machine, following a test procedure adapted from the ISO-10328:2016 proof load test procedure. User testing was performed with three able-bodied participants walking on a hip prosthesis simulator to evaluate level-walking ability. Outcomes showed that user stride parameters were mainly influenced by the fixed gait pattern programmed into the IHK control system. The results also showed that users chose different gait strategies that differed in prosthetic stance times and hip extension angles.

Future research includes completing the development of an adaptive control system and optimizing electronics that are shared between hip and knee joints, followed by user testing with hip amputee participants.

## Figures and Tables

**Table 1:** Example configurations of the adjustable interface and their corresponding interface and IHK lengths.

Adjustable Interface Components	Interface Length (cm)	IHK Length (cm)	Comments
Male four-hole pyramid adapter + female four-hole pyramid adapter	2.44	41.44	Shortest possible configuration using only two four-hole adapters. Only provides one pyramid connection for angle adjustment.
Female four-hole pyramid adapter + male–male double pyramid adapter + female four-hole pyramid adapter	4.32	43.32	Shortest configuration that provides two pyramid adapters for double angle adjustment.
Male four-hole pyramid adapter + pylon clamp with female pyramid adapter + pylon + four-hole pylon adapter	>9.64	>48.64	One pyramid adapter for angle adjustments. Pylons can be as long as necessary.
Male four-hole pyramid adapter + pylon clamp with female pyramid adapter + pylon + pylon clamp with female pyramid adapter + male four-hole pyramid adapter	>11.68	>50.68	Two pyramid adapters for angle adjustments. Pylons can be as long as necessary.
Male four-hole pyramid adapter + adjustable length pylon with female pyramid adapter ends + male four-hole pyramid adapter	9.39–11.19 11.14–14.54	48.39–50.19 50.14–53.54	Two pyramid adapters for angle adjustments. Provides a range of lengths without the need for custom length pylons. Össur adjustable pylons [23] come in two length options.

**Table 2:** ISO-10328:2016 P5 LCI test loads for static load tests.

Loading Test	LCI P5 Load (N)
Ultimate Static Load Test	4480
Proof Load Test	2240

**Table 3:** Test prosthesis assembly mass, including electronics and batteries.

HKAF Components	Mass (kg)
Hip simulator + HKAF	9.50
HKAF	6.25
IHK	5.00
Shank + ankle + foot	1.25

**Table 4:** Participant information.

Participants	Age (Years)	Height (cm)	Mass (kg)	Number of Walking Canes
A	44	178	95	1
B	28	180	95	2
C	26	175	98	2

**Table 5:** Average and standard deviation for stride parameters for each participant, for prosthetic and intact limbs. Percentages indicate each gait parameter in proportion to stride time.

Participants	Stride Time (s)	Step Time (s)		Swing Time (s)		Double Support Time (s)	Cadence (Steps/min)
		HKAF	Intact	HKAF	Intact		
A	2.41 ± 0.08	1.53 ± 0.11 (64%)	2.13 ± 0.08 (88%)	0.93 ± 0.05 (39%)	0.28 ± 0.02 (12%)	1.20 ± 0.12 (50%)	24.93 ± 0.82
B	2.51 ± 0.11	1.22 ± 0.11 (49%)	2.23 ± 0.07 (89%)	1.29 ± 0.06 (50%)	0.27 ± 0.01 (11%)	0.94 ± 0.07 (40%)	23.97 ± 1.06
C	2.50 ± 0.21	1.26 ± 0.04 (51%)	2.02 ± 0.14 (81%)	1.24 ± 0.08 (51%)	0.48 ± 0.06 (19%)	0.78 ± 0.07 (29%)	24.15 ± 2.05
Average	2.47 ± 0.14	1.34 ± 0.16 (55%)	2.12 ± 0.15 (86%)	1.15 ± 0.22 (46%)	0.35 ± 0.11 (14%)	0.97 ± 0.20 (40%)	24.35 ± 1.41

**Table 6:** Average and standard deviation for IHK hip and knee range of motion for each participant.

Participant	Max Hip Extension (°)	Max Hip Flexion (°)	Hip Range of Motion (°)	Max Knee Extension (°)	Max Knee Flexion (°)	Knee Range of Motion (°)
A	−1.23 ± 0.20	33.93 ± 1.01	35.16 ± 1.11	−1.87 ±0.02	30.98 ± 4.04	32.85 ± 4.02
B	−2.22 ± 0.29	31.60 ± 0.82	33.82 ± 0.80	−1.77 ± 0.05	57.42 ± 3.57	59.19 ± 3.58
C	−12.03 ± 0.23	28.70 ± 1.48	40.73 ± 1.34	−1.97 ± 0.04	38.23 ± 9.99	40.20 ± 10.01
Average	−5.16 ± 4.98	31.41 ± 2.44	36.57 ± 3.23	−1.87 ± 0.09	42.21 ± 13.00	44.08 ± 12.95

**Table 7:** Comparison between the IHK HKAF prosthesis and alternatives in the literature.

Category	IHK HKAF	Ottobock Helix3D + C-Leg *	Ottobock 7E7 + C-Leg **	HKAF by Ueyama et al. [15]
Hip joint type	DC motor	Four-bar linkage, polycentric mechanical joint with adjustable damping and two energy-return springs [18,29]	Monocentric mechanical joint with energy-return spring [18,30]	DC motor
Knee joint type	Magnetorheological variable-damping actuator	Variable hydraulic-damping actuator [7]	Variable hydraulic-damping actuator [7]	DC motor
Mass (kg)	HKAF: 6.25 IHK: 5.00	Hip: 0.99 [31] Knee: 1.25 [32]	Hip: 0.875 [33] Knee: 1.25 [32]	HKAF: 9.8
Availability	Research-only device	Commercially available	Commercially available	Research-only device

* Ottobock recommends the use of the Ottobock C-Leg or Ottobock Genium with the Helix3D hip. ** Ottobock does not provide recommendations for knee prosthesis use with the 7E7.

## Data Availability

Data sharing is not applicable to this article.
